# New Frontiers in Colorectal Cancer Treatment Combining Nanotechnology with Photo- and Radiotherapy

**DOI:** 10.3390/cancers15020383

**Published:** 2023-01-06

**Authors:** Sara C. Freitas, Daniel Sanderson, Sofia Caspani, Ricardo Magalhães, Belén Cortés-Llanos, Andreia Granja, Salette Reis, João Horta Belo, José Azevedo, Maria Victoria Gómez-Gaviro, Célia Tavares de Sousa

**Affiliations:** 1 IFIMUP-Institute of Physics for Advanced Materials, Nanotechnology and Photonics of University of Porto, LaPMET-Laboratory of Physics for Materials and Emergent Technologies, Departamento de Física e Astronomia, Faculdade de Ciências, Universidade do Porto, Rua do Campo Alegre s/n, 4169-007 Porto, Portugal; 2 Instituto de Investigación Sanitaria Gregorio Marañón (IiSGM), Doctor Esquerdo 46, 28007 Madrid, Spain; 3 Departamento de Bioingeniería e Ingeniería Aeroespacial, Universidad Carlos III de Madrid, 28911 Leganés, Spain; 4 Department of Medicine, Duke University, Durham, NC 27705, USA; 5 LAQV, REQUIMTE, Departamento de Ciências Químicas, Faculdade de Farmácia, Universidade do Porto, R. Jorge de Viterbo Ferreira 228, 4050-313 Porto, Portugal; 6 Colorectal Surgery—Champalimaud Foundation, Champalimaud Centre for the Unknown, Avenida Brasília, 1400-038 Lisboa, Portugal; 7 Departamento de Física Aplicada, Facultad de Ciencias, Universidad Autonoma de Madrid (UAM), Campus de Cantoblanco, C/ Francisco Tomas y Valiente, 7, 28049 Madrid, Spain

**Keywords:** cancer, colorectal, single-cell, phototherapy, radiotherapy, gold, nanoparticles

## Abstract

**Simple Summary:**

Although colorectal cancer is the third most common type of cancer, its treatment strategies still have room for improvement, as current techniques carry risks that aggressively deteriorate patients’ quality of life. Medical physics using nanotechnology tools can significantly contribute to solving this challenge. The high biocompatibility, easy functionalization, and targeting capabilities of plasmonic (gold) nanoparticles, together with their high atomic number, which provides a large X-ray absorption cross-section, makes them potential agents for enhancing cancer therapeutics as photothermal therapy agents and as radiosensitizers. Hence, the auspicious possibility of synergistically combining radio- and phototherapies is of imperative importance and must be explored to enhance their clinical application.

**Abstract:**

Colorectal cancer is the third most common cancer worldwide. Despite recent advances in the treatment of this pathology, which include a personalized approach using radio- and chemotherapies in combination with advanced surgical techniques, it is imperative to enhance the performance of these treatments and decrease their detrimental side effects on patients’ health. Nanomedicine is likely the pathway towards solving this challenge by enhancing both the therapeutic and diagnostic capabilities. In particular, plasmonic nanoparticles show remarkable potential due to their dual therapeutic functionalities as photothermal therapy agents and as radiosensitizers in radiotherapy. Their dual functionality, high biocompatibility, easy functionalization, and targeting capabilities make them potential agents for inducing efficient cancer cell death with minimal side effects. This review aims to identify the main challenges in the diagnosis and treatment of colorectal cancer. The heterogeneous nature of this cancer is also discussed from a single-cell point of view. The most relevant works in photo- and radiotherapy using nanotechnology-based therapies for colorectal cancer are addressed, ranging from in vitro studies (2D and 3D cell cultures) to in vivo studies and clinical trials. Although the results using nanoparticles as a photo- and radiosensitizers in photo- and radiotherapy are promising, preliminary studies showed that the possibility of combining both therapies must be explored to improve the treatment efficiency.

## 1. Introduction

Colorectal cancer (CRC) caused 1.9 million new cases and 0.9 million fatalities in 2020 [1]. As a result, CRC is considered the second most fatal cancer and third most common malignancy, whose prevalence is projected to alarmingly increase over the next years [1]. Considering population aging, growth, and human development, the latest GLOBOCAN data predict that the number of new CRC cases will reach 3.2 million in 2040 [1]. In high-income nations, the rising incidence of CRC is more prominent, partly attributable to lifestyle decisions, including eating habits and physical activity levels [1,2]. Despite the advances made in the last 40 years, namely, with the introduction of new drugs, radiotherapy, and the use of more accurate surgical procedures, the treatment of CRC continues to be extremely complex, with a survival rate that varies according to the stage of the disease at diagnosis and that usually ranges from 90% in patients with localized cancer to 14% in people with metastatic cancer [3]. Therefore, it is urgent to tackle this disease with different therapeutic strategies that may achieve better survival. A more personalized therapeutic strategy guided by new materials for early and accurate diagnoses and the ability to enhance tumor responses to treatment will probably be the way to achieve this goal [3,4]. The use of nanomaterials is currently a hot topic with a wide range of applications for medical diagnosis and treatment. Nanotechnology plays a major role in both the biotechnology and medical fields, as it offers new techniques to overcome the constraints of conventional medicine. The purpose of this article is to offer a comprehensive evaluation of the most relevant clinical and research findings regarding the use of nanoparticles (NPs) in CRC treatment. With a focus on gold nanostructures, it will address the application of NPs in radiotherapy (acting as radiosensitizers), nanoparticle-induced hyperthermia techniques such as photothermal therapy (PTT), and the synergistic effect that could arise from the combination of both treatments.

## 2. Challenges in Colorectal Cancer: A Medical Point of View

CRC is currently managed with a set of varied and advanced techniques that are adaptable to the patient and the characteristics of the neoplasm, including radiotherapy (RT), chemotherapy (CT), and surgery [2,5,6]. The staging of the tumor and its location are the most important criteria in determining which therapy is useful and how effective it may be [4,7]. The local extent of the tumor, its spread to nearby tissues, and the involvement of lymph nodes or distant sites (metastasis) are what determine the stage of the disease [4]. In its early stages, pre-cancerous lesions are confined to the inner layer (mucosa) of the colon or rectum (stage 0) [4,8]. As CRC progresses to stage I, the tumor outgrows the muscularis mucosa (thin muscle layer of mucosa) into the submucosa and into the muscularis propria. In stage II, the cancer has spread to the colon’s or rectum’s outermost layers but has not penetrated through them, and in stage III, it has progressed to the neighboring lymph nodes, vessels, or fat deposits surrounding them. In the highest and most aggressive stage (stage IV), CRC has progressed to distant organs, most frequently to the liver or lungs, or to distant parts of the peritoneum (the fascia covering the abdominal cavity) [4,8]. 

Surgery is frequently the first line of treatment for this cancer in its early stages (stages I-II), while chemotherapy is typically prescribed as the first option for metastatic CRC (stage IV) [9]. In the presence of risk factors for local recurrence, neoadjuvant treatments with the use of RT and CT, i.e., prior to surgery, are the gold standard [10]. In fact, recently, there has been an increase in the use of neoadjuvant treatments for rectal cancer, with the goal of achieving a complete tumor response. This increase has allowed patients to be treated with an organ preservation strategy, i.e., a non-surgical approach called watch-and-wait [10]. This strategy translates into a significant increase in the patient’s quality of life, who would otherwise be referred for surgical procedures with serious consequences, namely, on urinary, sexual, and defecatory functions, and, in many cases, the need for a permanent stoma [11]. However, a major concern in watch-and-wait patients is related to the risk of local regrowth after a complete clinical tumor response. About 30% of patients will require additional surgical procedures with a subsequent loss of quality of life [11]. Improvement is therefore essential on two fronts: (i) increase the response of colorectal cancer to neoadjuvant treatment to achieve higher rates of complete tumor response; (ii) more accurate and earlier diagnosis, in terms of both initial staging and post-treatment surveillance. The upward development of nanotechnology during the last few decades has been seen as a promising approach for cancer diagnosis and treatment, as several NP-based assays demonstrated improved selectivity and sensitivity with new capabilities not possible with the existing conventional techniques [3,4,12,13,14]. These advancements not only may improve cancer patient survival by allowing an early diagnosis but also might be used to track cancer progression in response to treatment [12]. This is particularly relevant for localized tumors, where early diagnosis and treatment allow a much higher rate of survival. 

Magnetic nanoparticles (MNPs), quantum dots (QDs), and gold nanoparticles (AuNPs) are some examples of nanomaterials that have already demonstrated the potential to facilitate the diagnosis and prognosis of different cancers, including breast cancer, lung cancer, leukemia, liver cancer, colon cancer, and rectal cancer [3,4,12]. When properly functionalized with the right ligands, QDs can act as biomarkers and recognize specific cancer-associated molecules to allow their visualization by different imaging techniques [15,16]. In parallel to this, iron oxide nanoformulations (MNPs) and AuNPs, taking advantage of their physical properties, can also act as contrast agents in imaging techniques such as magnetic resonance (MRI) and computed tomography [17,18,19]. Beyond their diagnostic capabilities, such NPs can also have an active role in cancer treatment. The utilization of nano-based carrier systems as a means of drug delivery is one of the most promising cancer therapy methods [14,19]. In parallel to this, the strong photoelectric absorption coefficient of gold (its high Z (Z = 79)) provides a large X-ray absorption cross-section that, when combined with high Auger and Coster-Kronig (C-K) electron emission yields, makes AuNPs excellent radiosensitizers, offering significant local radiation dose enhancement [20]. The results show that using nanoparticles in cancer diagnostics and treatment is a promising approach to combating the current difficulties and limitations, unlocking new frontiers and treatment modalities.

## 3. Heterogeneity in Colorectal Cancer: Single-Cell Point of View

Intratumoral heterogeneity is likely to have implications for cancer treatment, local regrowth, and biomarker discovery, particularly for targeted treatments. In the case of CRC, it presents complex and heterogeneous phenotypes with effects at the epigenomic, genomic, transcriptomic, proteomic, and metabolomic levels [21,22]. Single-cell technologies have contributed to exciting progress in understanding the single-cell heterogeneity of carcinogenesis progression and metastasis, which can be fundamental to knowing the tumor in depth and choosing the most appropriate treatment [23,24]. CRC heterogeneity studies focus on clonal heterogeneity, the microenvironment, spatial organization and crosstalk, cell differentiation, metastasis, the therapy resistance of human T cells, non-immune cells, organoids, in vivo models, and patients [25,26,27]. Given the complexity of CRC and tumor heterogeneity, it is important to integrate patient information into models to predict heterogeneous cancer evolution [28]. Organ-on-a-chip 2D and 3D models have been developed by culturing primary human intestinal epithelial stem cells to mimic and shape the human intestinal structure and mechanical properties [28,29,30,31]. Three-dimensional in vitro platforms have shown that 90% of genetic mutations in patient tumors are present in tumor organoids, corroborating the robust use of organoids to study tumor heterogeneity (as discussed in detail in Section 6.2) [32,33]. The culturing of organoids from metastatic colorectal cancer patients’ biopsies has a success rate of 71%, revealing a new tool for the clinic as a potential platform for treatment decisions for the individual patient [33].

**Figure 1:**
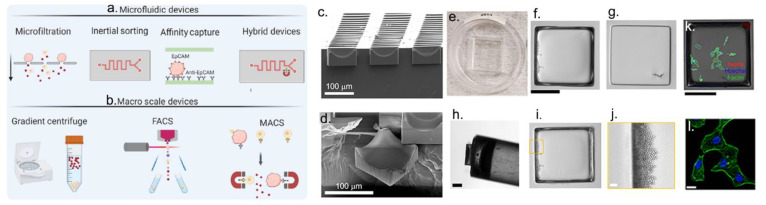
Single-cell separation, sorting and collection (**a**) micro- and (**b**) macro-technologies. Reprinted from Ref. [49] with permission from Elsevier. (**c**) Example of a microarray device. Embedded microstructures arrays (EMAs): scanning electron microscopy cross-section images of a full array (**c**) and one microcarrier (**d**). (**e**) Transparent EMA glued to the cassette where cells will be seeded. (**f**–**j**) Since iron oxide microbeads were embedded into each microcarrier, magnetic collections were performed by a microwand to isolate cells and release them into a different container. (**k**,**l**) High-quality images of cancer cells on EMAs. Black scale bars: 250 µm; white scale bars: 20 µm. Adapted with permission from Ref. [37]. Copyright 2022, Creative Common CC BY.

Different single-cell technologies have been developed to study the morphology, plasticity, phenotype, genomics, transcriptomics, and proteomics of tumor heterogeneity [34]. Depending on the study and the sample type, high-throughput technologies have been developed, such as fluorescence-activated cell sorting (FACS), magnetic-activated cell sorting (MACS), microfluidics, and microarray devices (Figure 1) [35,36,37,38,39]. Innovation in high-spatial- and high-temporal-resolution imaging with the ability to make a sort decision is receiving attention to tie cell phenotypes to omics data [35]. Microarray technologies provide high spatial and temporal resolution, providing different approaches to collecting single cells for further genetic analysis [35]. Magnetic collections using microarrays can be accomplished when iron oxide microbeads or nanoparticles are embedded into the individual microcarrier [35,37]. Microarrays have been used for high-quality imaging and studying metastatic tumor cell motility and invasion over time [35,37,40]. Gracz et al. studied how a single intestinal stem cell (ISC) developed into an organoid over time by time-lapse imaging on a microarray platform. Moreover, quantitative polymerase chain reaction (qPCR) was performed to correlate the phenotype with gene expression analysis results. This study observed high levels of active ISC markers at 0 h, while secretory progenitor markers and differentiated cell markers were found at later time points, finally obtaining mature organoids after 240 h [41].

Technologies that provide a high throughput are ideal for large-scale multi-omics data classification to comprehensively characterize CRC at a single-cell level [42]. Among the different single-cell omics technologies, single-cell RNA sequencing (scRNA-seq) is receiving attention because it provides a full transcriptome characterization of single-cell diversity and heterogeneity. scRNA-seq measurements were used to gain insights into the CRC microenvironment composition, revealing the dynamic nature of immune and non-immune cells from CRC at different stages [43,44]. A single-cell triple omics sequencing (scTrio-seq) technique has been developed to characterize multiple layers of molecular features assessing simultaneity, somatic copy number alterations (SCNAs), DNA methylation, and transcriptome information from single cells. This technique has been used in CRC patients, showing that molecular alterations are important in metastases and cancer progression [45]. Different studies have observed molecular phenotype diversity and different mutations in transcriptome profiles from scRNA-seq measurements in colorectal organoids [46,47]. Single-cell DNA sequencing (scDNA-seq) and scRNA-seq techniques have been used to study drug resistance and intratumoral genetic diversity in organoids [48]. This study supports the strength of the characteristics of organoid clone cultures from individual cells. All colorectal cancers in this study presented resistance to the most common drugs used to treat the disease, which highlights the need for new treatments. Moreover, comparing the DNA sequences, they found a greater somatic mutation rate in CRC than in normal colorectal cells [48]. 

In summary, cell heterogeneity in cancers is one of the greatest challenges in enhancing cancer diagnosis and treatment for the development of effective personalized therapies. Single-cell studies of nanotechnology-based treatments using radiotherapy and phototherapy are also needed to understand the mechanism of interaction between radiation and nanoparticles from a single-cell point of view. Since CRC presents heterogeneous clinical behavior and diverse responses to treatment, the power of single-cell technologies and the creation of new model systems can address the complexity of CRC by developing improved and new strategies for treatment.

## 4. Nanoparticles in Radiotherapy

**Figure 2:**
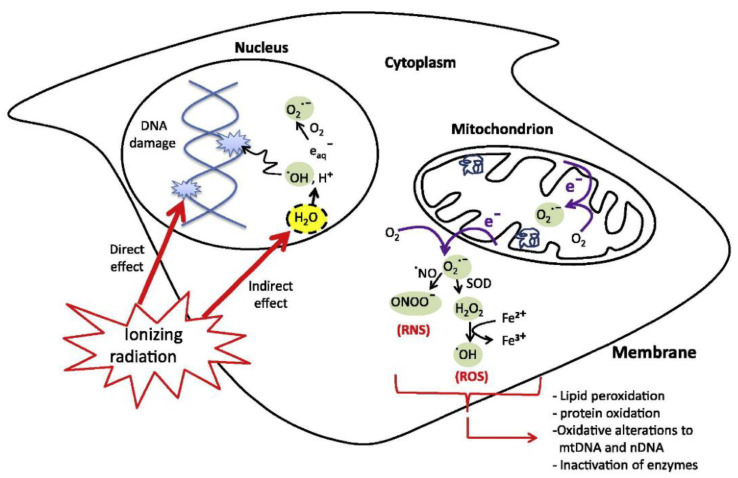
Direct and indirect cellular effects of IR. Reprinted with permission from Ref. [75]. Copyright 2022, American Chemical Society.

Radiotherapy (RT) is one of the most effective and widely used techniques for cancer treatment, and it is used in approximately 50% of cancer patients [50,51,52]. The mechanism behind this type of treatment is cellular damage caused by the interaction between ionizing radiation (IR) and biological tissues [53]. Radiation can be delivered to cancer cells either via an external beam (conventional RT) or through an internally implanted radiation source (brachytherapy) [54]. In external beam radiotherapy, the most frequently used therapies rely on the use of X-rays, where orthovoltage (100 to 500 keV) or megavoltage (>1 MeV) photons are commonly used to treat superficial or deep-seated tumors, respectively. Alternatively, energetic particles can also be employed, though they are less common due to their higher costs [55]. Cell exposure to IR triggers a cascade of processes that can be divided into three phases: physical, chemical, and biological [56,57,58]. The physical phase, which occurs in the first nanoseconds of irradiation, describes the interactions between charged particles and the atoms of which the tissue is composed. IR mainly interacts with orbital electrons, ejecting some of them from atoms (ionization) and raising others to higher energy levels (excitation). If the ejected secondary electrons are energetic enough, they may excite or ionize other surrounding atoms, thus triggering a cascade of ionization events. Because of the deposited energy and fast relaxation processes, various ionized/excited water molecules are generated (water radiolysis), resulting in the breakage of chemical bonds and the formation of free radicals, which are highly unstable. These water radicals, usually called reactive oxygen species (ROS), can be stabilized by other molecules in the medium through oxidation-reduction reactions. The ensemble of processes describing the mechanism of action of free radicals is usually referred to as the chemical phase. Besides the previously described reactions, ROS interact with different biological systems, such as DNA, lipids, and proteins. It is important to note that DNA damage can occur either through the so-called “indirect effect”, caused by ROS, or the “direct effect”, in which DNA is directly injured by the ionization-excitation processes that take place in the physical stage [59,60], as presented in Figure 2. Finally, the biological phase includes a series of cellular processes that are activated to repair radiation-induced damage [61]. Unrepaired damage can possibly result in malignant cell transformation or cell death over a span of seconds to days, or even years [62,63].

As already mentioned, IR damages multiple intracellular components. Despite being effective in damaging tumor cells, radiation therapy presents some critical drawbacks, such as dose heterogeneity, cell radioresistance, and damage to the surrounding healthy tissues. Consequently, the appropriate control of the delivered radiation dose is a crucial aspect to minimize toxicity to normal tissues [64]. To achieve more efficient dose delivery to targeted organs, numerous improvements in RT have emerged in the last few decades, such as intensity-modulated radiation therapy (IMRT), image guidance, stereotactic radiation therapy, and particle therapy [65,66,67]. However, increasing the maximum dose accumulation in cancerogenic tissues while sparing healthy ones remains a great challenge [62,68]. During the last several years, aiming to develop more effective anticancer regimes, a growing community of researchers has been exploiting the unique properties of nanomaterials to resolve the unsolved challenge of classical RT [69,70]. Among nanomaterials, nanoparticles (NPs) have been found to offer unique opportunities for RT [71,72,73] due to their specific properties, such as a high surface-to-volume ratio, enhanced cellular uptake, and ease of surface modification. Moreover, NPs are found to undergo deep tissue penetration, which increases the enhanced permeability and retention (EPR) effect [72]. In the context of radiotherapy treatment, NPs can either be used as radiosensitizers for external radiation beams, such as high-Z elements, or be employed as delivery formulations for therapeutic radionucleotides [55,74].

The concept of radio-sensitization dates to the mid-1970s, when enhanced radiation damage to chromosomal DNA was first reported in patients undergoing iodine angiography [76]. Later, it was discovered that metal implants could alter the delivered doses during radiation therapy treatments [77,78], and in the last decades, several studies have exploited the capability of nanosized high-Z metal particles in promoting dose delivery to cancer tissues as an opportunity to improve RT procedures [75]. In this context, the rationale for using high-Z materials as radiosensitizers was first based on differences in the energy absorption properties of metals compared to soft tissues, i.e., physical interactions. Depending on the nature and energy of the ionizing source, such as X-rays, protons, neutrons, and ions, different mechanisms can occur. When X-rays impinge on a material, photons can be attenuated by four major processes, namely, the photoelectric effect, Compton effect, pair production, and Rayleigh scattering [79], as presented in Figure 2. 

**Figure 3:**
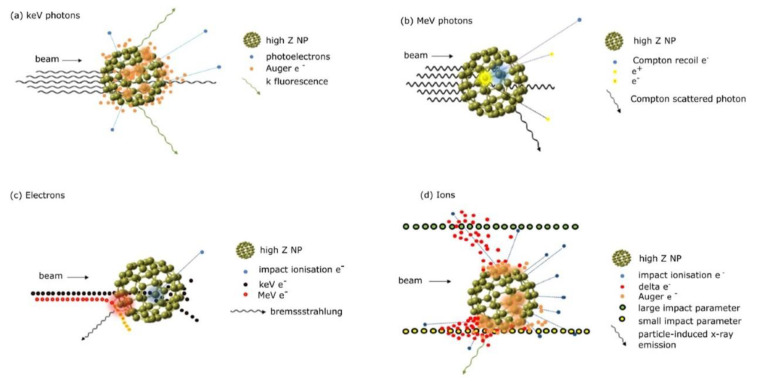
Mechanisms of interaction of high-Z NPs with (**a**) keV photons, (**b**) MeV photons, (**c**) electrons, and (**d**) ions. Reprinted with permission from Ref. [82]. Copyright 2022, Creative Commons.

In a low regime of ionizing radiation (<500 keV), the photoelectric effect is the dominant interaction process with high-Z atoms (Z = 60–80) [80,81,82,83]. In this framework, the photoelectric cross-section strongly depends on $Z^{3}/E^{3}$, allowing high-Z NPs to transfer radiation to the medium by photoabsorption and subsequent electronic emission more efficiently than water. In this process, the energy of the incident photon is completely absorbed by the atom, which ejects an electron from its inner shell, also called a photoelectron, which possesses kinetic energy equal to the energy of the primary beam minus its binding energy. To fill the generated inner vacancy, two possible situations can occur, resulting either in the production of characteristic X-rays (also called fluorescent photons or secondary radiation) or in the emission of an Auger electron [84]. Fluorescent photons can travel longer ranges, while Auger electrons travel short distances, being effective in producing very high local ionization density [74,85]. Since the PE has a relatively small contribution to absorption in soft tissues (where the Compton effect dominates), photoelectrons, secondary photons, and Auger electrons emitted from high-Z metal NPs will cause a highly localized dose enhancement in the surrounding cells as illustrated in Figure 3 [55,75,82]. To enhance the biological effects through physical mechanisms, the NPs must be in the proximity of the selected target. 

According to theory, NP-enhanced radiotherapy would only be effective with low-energy X-rays. At the same time, no increase in the overall dose deposition would be expected using higher energy or particle radiation, where the interactions between NPs and matter are dominated by the Compton effect, which does not strongly depend on Z [86,87]. However, radiosensitization with high-Z materials has also been observed for clinical MeV radiation [88,89,90,91], proton [92,93], and ion sources [94], indicating that the physical mechanism is not exhaustive in explaining the dose enhancement caused by the presence of such NPs [95,96]. In this regard, several studies have pointed out that other non-physical mechanisms, such as chemical and biological processes, play an important role in producing indirect damage to cancer cells. In fact, the main chemical effect contributing to NP-induced radiosensitization is the formation of ROS. ROS enhancement can also be produced by catalytic processes, apart from the physical effects previously described, which can result in oxidative stress, the interruption of the cell cycle, or the inhibition of DNA repair [97,98]. In this context, several studies have demonstrated that boosted ROS production can also be achieved by using metal oxide NPs with lower atomic numbers (made by Si, Al, Ti, Zn, Fe, and Ce, for instance) because of the high catalytic activities of their surfaces [99].

Over the past few years, NPs have been studied for their use as radiosensitizers to improve the response of cancerogenic tissues to radiotherapy procedures. In this framework, there has been considerable interest in the study of metallic, bimetallic, and metal-oxide-based NPs [100]. The most promising types of NPs are briefly described in the following paragraph. 

Among metallic NPs, gold nanoparticles (AuNPs, Z = 79) have been widely studied for either diagnostic or therapeutic applications in cancer therapy due to their high atomic number, good biocompatibility, and strong photoelectric absorption. Regarding RT, the increase in the local radiation dose in the presence of AuNPs is attributed to strong photoelectric interactions because of their high atomic number, but also to the generation of ROS when the NPs are irradiated with particulate/high-energy X-rays [89]. Moreover, the efficiency of Au NPs as radiosensitizers depends not only on the NP size, shape, concentration, and coating but also on the external beam energy and specific cell line [68,87,101]. Ongoing research is focusing on the optimization of NP characteristics to reduce the delivered doses and to combine IR with other treatment modalities [75,102]. Another candidate as a sensitizer is gadolinium (Gd, Z = 64), which is commonly employed as a magnetic resonance imaging (MRI) contrast agent. Studies on Gd-based NPs have confirmed a significant sensitization effect in the presence of ionizing radiation under different conditions, i.e., NP dimensions, coatings, beam energies, and cell lines, allowing AGuIX, a Gd and Si-based NP, to enter clinical trials. Moreover, due to their properties, Gd-based NPs have been considered suitable for use in combination therapies, such as image-guided RT [90,103]. Among metal-based NPs, silver NPs (Ag, Z = 47) have also attracted attention due to their enhanced radiation sensitization [104]. However, their ability to accumulate in tumor cells remains to be improved [100]. As an alternative to full metallic systems, metal oxide NPs have been considered a viable option. In this regard, Hafnium (Hf, Z = 72) oxide NPs (NBTXR3) have been considered an appropriate RT sensitizer because of their high atomic number, electron density, and chemical stability. Moreover, clinical trials concerning different tumor locations, such as head and neck, prostate, and rectal cancer, are being successfully carried out [80]. Besides the aforementioned NPs, other elements, having either high or low atomic numbers, may be employed as radiosensitizers, such as Fe, Ta, Bi, and Ce, for instance [75,82,100,105]. 

Moreover, although NPs are currently widely studied to directly enhance radiation effects, NPs can also be employed as delivery vehicles for tumor-specific radiosensitivity drugs due to their ability to be simultaneously functionalized with chelators and targeting agents, thus enhancing cellular uptake [55].

In the specific context of RT for colorectal cancer, Au, Ag, and SPIO NPs have shown promising results as radiosensitizers (which will be discussed in detail in Section 6) [5,106,107,108]. In addition to this, several types of NPs are being explored for their use as nanocarriers for targeted therapy, which can be combined with RT to achieve better results [109,110,111]. However, it is worth mentioning that despite the large number of studies, there are clear difficulties in translating preclinical studies into clinical trials. These adversities may be related to the large number of variables that must be investigated to control and optimize the desired outcome. The reported studies have examined different aspects, such as diverging cell lines, NP materials and coatings, and radiation parameters. Consequently, experimental data reporting the radiation enhancement effects produced by several types of NPs on different biological systems have shown large variability. Hence, despite the promising preclinical results of NP-mediated radiosensitization, the exact mechanism of the interaction between NPs and ionizing radiation, as well as the subsequent biochemical and biological effects, remains incomplete, and further studies are therefore required, namely, studies using single-cell models, as discussed in the previous section [80,112,113].

## 5. Nanoparticle-Based Photothermal Therapy

A potential oncologic therapy method known as hyperthermia involves heating cancer cells up to 40–45 °C to induce apoptosis, or programmed cell death [19,114]. The traditional hyperthermia method, involving an external heating source that generates a temperature gradient to a maximum on the body’s surface that rapidly decreases with distance, has several drawbacks, not only because the energy is dissipated in healthy tissues located between the surface and the tumor but also because there is no differentiation between the targeted tissue and the surrounding normal tissues [115]. The advent of nanotechnology and its recent developments enable these drawbacks to be tackled by providing an appropriate way for localized and differentiated heat delivery to reach local and controlled hyperthermia. This is achieved via functionalized nanostructures that are activated by external/outside stimuli, such as electromagnetic radiation. In this context, functionalized nanoparticles, when concentrated inside the tumor, can absorb the energy coming from the external source to locally enhance the effects of hyperthermia [19,115]. Photothermal therapy (PTT) is an excellent example of innovative nanotechnology-based strategies. It is a nanoparticle-mediated hyperthermia technique that consists of a minimally invasive localized treatment, whose goal is to convert electromagnetic radiation into heat by stimulating photoabsorbing agents that are administrated to the body intravenously or intratumorally [19,114]. Laser light in the near-infrared (NIR) region is the energy source typically used in PTT due to its enhanced tissue penetration capability, with lower absorption in biological tissues [19,116]. In particular, the highest transmittance for NIR is found in tissue components such as hemoglobin and water, which allows NIR radiation to go through 10 cm of subcutaneous tissue, 4 cm of skull/brain tissue, or 4 cm of muscle tissue [116]. Noble metal NPs, carbon-based nanomaterials, metal compounds, and organic nanomaterials are some of the types of photothermal agents currently under development for application in PTT [116]. Among them, gold nanostructures are the most popular and have been extensively explored due to their biocompatibility, versatility, and high light-to-heat conversion efficiency and the fact that by controlling their shape and aspect ratio, it is possible to tune their absorption spectra peaks to match the desired wavelength, including the maximum absorption in the NIR region for optimal tissue penetration [2,19]. In this case, the heat generation phenomenon can be explained by a feature of metallic nanoparticles called surface plasmon resonance (SPR) [19,114,117]. SPR is caused by the presence of conduction electrons that oscillate on the metallic nanoparticle surface. If the incident light wavelength/frequency triggers the resonance of the oscillating electrons, a high-energy state is reached, leading to sequential energy dissipation in the form of heat. Under these circumstances, the light absorption results in optimal heat generation at the NPs that ultimately dissipates from the particle to the surrounding media [19,114]. 

Phototherapy mediated by gold nanoparticles has already shown promising results in vitro and in vivo (with animal models) for different types of cancer, including breast, prostate, lung, colon, and colorectal cancer. Manivasagan et al. demonstrated that gold nanorods ([AuNR] = 25 µg mL^−1^), duly modified for biocompatibility purposes, combined with laser irradiation (2 W cm^−2^, 5 min) induced significant apoptosis (63.3%) when compared with three control groups (0.38%, 1.74%, and 12.01%) in breast cancer cells (MDA-MB-231) [118]. Sangnier et al. studied the photothermal effect of four gold nanoparticle morphologies and the resulting induced cancer cell death on human prostate cancer (PC3) cells. For cancer cells exposed to 808 nm NIR irradiation (0.3 W cm^−2^, 10 min) with gold nanorods ([AuNR] = 98.5 µg mL^−1^), the number of viable cancerous cells decreases to 38% in the extracellular condition and to 11% in the intracellular situation [119]. Taking advantage of the ability to functionalize NPs to target specific cancer types, Knights et al. studied the use of pulsed and continuous lasers operating at 854 nm on lung cancer cells (A549) with nanorods functionalized with anti-EGFR antibodies. It was demonstrated that pulsed laser irradiation (pulse repetition frequency: 10 Hz; pulse duration: 7 ns; spot size: 9 mm; radiant exposure: 25 mJ cm^−2^) resulted in a 93% ± 13% reduction in cell viability when compared to control exposures, highlighting the efficiency of this minimally invasive therapy [120].

Specifically considering CRC, in 2010, Goodrich et al. evaluated the efficacy of photothermal treatment in a murine subcutaneous colon cancer model (CT26) as summarized in Table 1. Here, the solid tumors were infused with PEGylated gold nanorods, followed by the percutaneous irradiation of the tumor with an 808 nm laser (3.5 W, 180 s exposure time). Statistics show that the photothermally treated group’s survival outlasted that of the control groups, and 60 days after the treatment, 44% of the nanorod-treated mice survived with evidence of complete tumor ablation [121]. Three years later, Kirui et al. report the use of immunotargeted hybrid gold-iron oxide NPs for PTT treatment in a xenograft colorectal cancer tumor model (SW 1222 cells in subcutaneous-tumor-bearing mice) [122]. Here, PTT-induced toxicity (808 nm; 5 W/cm^2^, 20 min every 48 h) was verified by histological assessments of treated tumors, which showed over 65% necrosis in positive controls after 14-day photothermal therapy. In addition, this kind of NP can be used simultaneously as a contrast agent for non-invasive MR imaging: the gold NP portion of NPs works as an hyperthermia agent, while the iron oxide portion acts as an MR imaging agent [122]. Continuing the dual-function theragnostic NP trend, Azhdarzadeh et al. used gold-coated SPIONs (superparamagnetic iron oxide nanoparticles) functionalized with thiol molecules for the MRI and PTT of colon cancer cells (HT-29 cells). They observed that after irradiation (LED, 820 nm, 0.7 W/cm^2^, 2–8 min), a remarkable 80% of the cancerous cells were dead ([Au@SPIONs = 500 μg/mL]). The harmlessness of this type of irradiation was also proven by the almost null effect verified on the viability of NP-free cells [123]. In 2018, Chen et al. used gold nanorods conjugated with carbonic anhydrase IX (an antibody that specifically binds to a biomarker of hypoxia) to facilitate the targeting of hypoxic tumor areas. In vivo tests on subcutaneous HT29 xenografts revealed that the irradiation (760 nm, 12 W/cm^2^, 2–3 min) of mouse tumors injected with GNR/anti-CAIX led to complete ablation without tumor regrowth [124]. More recently, Simón et al. reported the effect of silica-gold nanoshells (NS) injected in a murine tumor model (CT26). Here, with fractionated PTT (807 nm, 1.2 W/cm^2^, 5 min) significant reductions in tumor sizes were found, and even complete regression was observed [125]. They also reported no significant changes in tumor development or survival between the groups receiving single-dose PTT and fractionated PTT.

Assembling all of these findings, it is noticeable that the most often utilized in vivo model in preclinical studies is the induction of subcutaneous tumors, with CT26, C26, and HCT116 being the most used CRC-related cell lines in BALB/c mice [126]. It is also apparent that metallic nanoformulations are quite popular, and, in several cases, they are also suitable for theragnostic and imaging purposes. Regarding PTT and laser parameters, each group has its own protocol, which makes comparing the effectiveness of NPs for heating a challenge. The only common parameter is that most preclinical studies operate in the first NIR biological window (700–950 nm). Still, different metal concentrations are utilized, power densities range from 0.1 to 6 W/cm^2^, and irradiation intervals range from a few seconds to minutes [127].

Over the years, PTT has proved its potential to selectively ablate malignant tissue and has been performed successfully on animals with small tumors. However, larger and more clinically significant tumors have been found to be more challenging in their complete removal without recurrence [125,128,129]. One of the main causes of this is that larger tumors frequently have hypoxic zones with poor blood flow, which, along with high interstitial pressure, hinders the transport of nanoparticles. In addition, the uneven nanoparticle distribution and the limited laser penetration depth can lead to heterogeneous intratumoral heat distributions [125]. These are challenging obstacles to overcome, but researchers have been inspired to investigate alternative uses of PTT in combination with other therapies, such as boosting the effectiveness of chemotherapy, releasing anticancer drugs, or inducing an immunological response for immunotherapy [19,130,131,132]. In addition to this, NPs can be exploited as radiosensitizers (Section 4), offering a significant enhancement of radiation damage to tumor tissue with reduced side effects on healthy surrounding cells [133,134,135]. Although not related to colorectal cancer, it is worth mentioning that gold nanoparticle systems have already moved to PTT clinical trials. In this context, AuroLase (photoabsorber-conjugated gold-silica NPs) is being developed for cancer therapy against head and neck (NCT01679470), lung (NCT00848042), and prostate tumors (NCT02680535) [136,137,138].

## 6. Photo- and Radiotherapy for Colorectal Cancer Treatment

### 6.1. In Vitro Studies

In vitro studies are fundamental for the proof-of-concept of the therapeutic potential associated with any treatment modality. Consequently, it is crucial to perform these assays for the clinical implementation of new strategies for treating colorectal cancer, namely, the combination of photothermal therapy (PTT) and radiotherapy. A search through the literature reveals that this theme has not yet been addressed and, therefore, represents an eventual new path for future research. Nevertheless, different authors have evaluated the suitability of PTT alone (Section 5) or in combination with chemotherapy, i.e., chemo-photothermal therapy, for treating this type of cancer using different nanomaterials [5,139,140,141]. Promising results were obtained due to the synergy of different treatments, as discussed in this section, which opens a window for future studies combining radiation techniques. 

Once again, one example concerns nanomaterials composed of noble metals, such as AuNPs, since it is possible to take advantage of their tunable surface plasmon resonance properties to produce localized photothermal heating [116]. In this context, Hosseinzadeh et al. analyzed the potential of a chemo-photothermal strategy in vitro using SN38-conjugated hyaluronic acid AuNPs in two colon cancer cell lines (HT-29 and SW480) [132]. Here, the authors were able to reduce cell viability to below 10% after irradiation with red light. However, such nanomaterials present an absorption peak in the visible range and, therefore, are not adequate for in vivo applications. 

This problem was overcome by White et al., who hybridized AuNPs with ultrasmall superparamagnetic iron oxide nanoparticles, obtaining hybrid magnetic AuNPs with an absorption peak in the near-infrared region (NIR). These nanoparticles were conjugated with a monoclonal antibody that targets CC-531 cells, causing a reduction in cell viability of up to 83% after exposure to NIR radiation [142]. Kirui et al. also produced Au and iron oxide hybrid nanoparticles, having analyzed their suitability for PTT in two colon cancer cell lines (SW1222 and HT-29). These nanomaterials were biofunctionalized with an antibody that targets those cells, and, after NIR irradiation, it achieved a maximum cell death of 65% [143].

**Figure 4:**
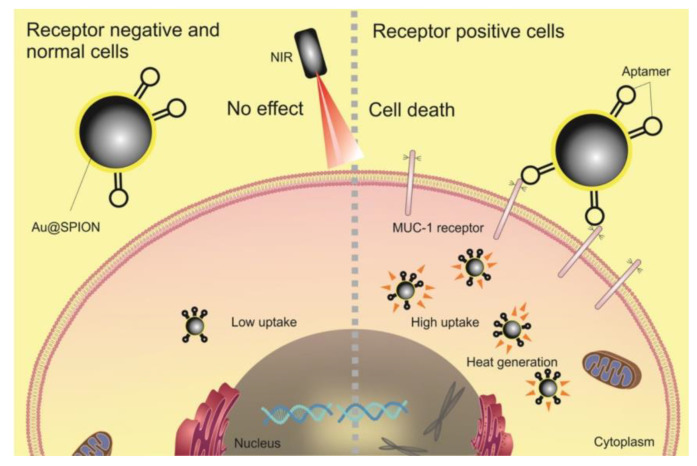
MUC-1 aptamer-targeted gold-coated superparamagnetic iron oxide nanoparticles for PTT and MRI of colorectal cancer. Reprinted with permission from Ref. [123]. Copyright 2022, Elsevier.

An alternative way to tune the absorption peak of Au nanomaterials is by modifying their aspect ratio, resulting in elongated nanostructures. In this context, Seo et al. fabricated methylene blue-loaded Au nanorods with a SiO_2_ shell, having assessed their suitability for a dual-treatment strategy for colorectal cancer involving photothermal and photodynamic therapy [144]. Here, the authors used the CT26 cell line, and, after a NIR laser irradiation of the nanoparticle-loaded cells, they observed a reduction in cell activity to 11%. Guo et al. also studied this type of nanomaterial and produced fluorescein isothiocyanate/cisplatin-loaded chitosan-Au nanorods and evaluated their potential as chemo-photothermal and contrast agents for real-time cell imaging [131]. Through the use of the LoVo cell line, these authors achieved considerable cell death under NIR irradiation and, simultaneously, optically imaged these cells. Another noble metal nanomaterial suitable for PTT is Au nanoshells, which present an absorption peak that can be tuned by adjusting their size and thickness. In this context, Azhdarzadeh et al. synthesized MUC-1 aptamer-targeted Au-coated superparamagnetic iron oxide nanoparticles and verified that these nanomaterials were able to not only work as contrast agents in MRI but also reduce the HT-29 cell viability upon NIR irradiation (Figure 4), working, therefore, as a theragnostic agent [123]. Regarding theragnostics, Wang et al. also reported the use of assembled phage fusion protein-modified Au-Ag hybrid nanorods for PTT and fluorescence imaging [145]. Here, in vitro studies were performed with SW-620 cells, having verified that it was possible to acquire fluorescence images of the cells and, simultaneously, reduce their viability to 30% through irradiation with a NIR laser. 

**Figure 5:**
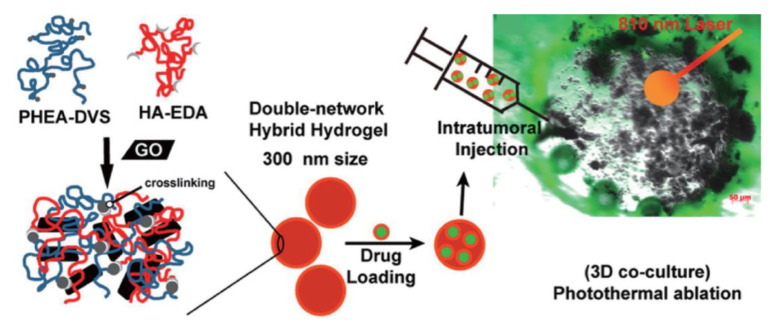
Drug-loaded graphene oxide nanogels for destruction of HCT-116 cells. Reprinted with permission from Ref. [146]. Copyright 2022, American Chemical Society.

A different type of nanomaterial that also presents an absorption capacity in the NIR region are those based on carbon, such as graphene and carbon nanotubes. In this context, Fiorica et al. demonstrated that it was possible to load graphene oxide nanogels with a chemotherapeutic drug, having assessed their potential for chemo-photothermal therapy using HCT-116 cells (Figure 5) [146]. As a result, the authors observed that, after NIR irradiation of the cells incubated with the nanogels, the cell viability dropped to almost 0%. A different work by Einafshar et al. addressed the fabrication of SN38-conjugated cyclodextrin-coated graphene oxide for chemo-photothermal therapy, as well [147]. Here, the authors reduced the HT-29 cell viability by ~14% after the exposure of cells incubated with this nanomaterial to a NIR laser.

The potential of multiwalled carbon nanotubes for this biomedical application has also been studied by some authors, such as Levi-Polyachenko et al., who analyzed their suitability for hyperthermic chemotherapy delivery using RKO and HCT-116 cells [148]. Here, it was verified that the NIR irradiation of these cells in the presence of multiwalled carbon nanotubes and oxaliplatin led to a significant reduction in their viability. In a different work, Graham et al. produced folic acid-functionalized multiwalled carbon nanotubes and assessed their potential for the PTT of colorectal cancer, considering the same cell lines [149]. As a result, the authors verified that the functionalized multiwalled carbon nanotubes presented 400% to 500% higher affinity for these cells compared to the non-functionalized ones. Additionally, after NIR irradiation, they observed a reduction in cell viability between 50% and 60%. Regarding this theme, Tan et al. produced poly (carbonate-urea) urethane-functionalized multiwalled carbon nanotubes for the thermal ablation of cancer cells through exposure to NIR radiation [150]. This study was performed using HCT-116 cells and, after performing the treatment, achieved a reduction in cell viability of 95.12%.

Besides nanomaterials based on Au and carbon, metal compound nanomaterials have also been studied in the context of colorectal cancer treatment. One example is the work by Koo et al., who fabricated nanocomposites of copper sulfate and assessed their suitability for the in vitro PTT of Caco-2 cells [151]. Here, it was observed that the NIR irradiation of nanocomposite-incubated cells led to a reduction in cell viability as high as about 80%. In another work, Hessel et al. produced amphiphilic polymer-coated copper selenide nanocrystals, which presented a strong absorption in the NIR region, and assessed their potential for the PTT of colon cancer in vitro using the HCT-116 cell line [152]. As a result, the authors verified that, after NIR irradiation, all cells exposed to the fabricated nanocrystals were destroyed. 

The suitability of organic nanomaterials for this biomedical application has also been investigated by a few authors. For example, Obiweluozor et al. synthesized polydioxanone nanofibers containing polydopamine nanospheres and bortezomib for chemo-photothermal therapy using the CT-26 cell line [153]. In this study, after NIR irradiation of the nanofiber-incubated cells, there was a verified reduction in cell viability to 5%. Another work by Kelkar et al. evaluated the use of nanoparticles based on a semiconducting conjugated polymer known as poly-[4,4-bis(2-ethylhexyl)-cyclopenta [2,1-b;3,4-b′]dithiophene-2,6-diyl-alt-2,1,3-benzoselenadiazole-4,7-diyl] (PCPDTBSe) in causing cell death through heat generation resulting from irradiation [154]. The authors verified that this nanomaterial possessed a dual absorption in the electromagnetic spectrum’s blue and NIR ranges. Subsequently, they were used in PTT tests with CT-26 cells, achieving a 90% reduction in cell viability. In another study, MacNeill et al. reported the use of a different organic nanomaterial, i.e., low-band-gap donor-acceptor-conjugated polymer nanoparticles, for the PTT of two different colorectal cancer cell lines (RKO and HCT-116) [155]. In this study, the authors achieved a cell viability lower than 10% after irradiation with a NIR laser.

In conclusion, it is verified that various authors have addressed the use of multiple nanomaterials for the PTT or chemo-photothermal therapy of colorectal cancer in vitro, having achieved promising results with different cell lines. Nevertheless, the combination of PTT with radiotherapy for the treatment of this specific cancer has not yet been reported, and, therefore, is a new research path that should be explored in the future.

### 6.2. Three-dimensional Models

Two-dimensional cultures are useful tools in cancer therapy to study the toxicity and efficacy of different treatment modalities in a reproducible and quick manner. Nevertheless, due to their simplicity, the data obtained with these models may not correlate with subsequent in vivo and clinical studies [156]. In this context, 3D models represent a more realistic approach to studying the outcomes of different therapeutics, including nanomedicines, radiotherapy, and photothermal therapy [157,158], contributing to the reduction in the number of animals when proceeding to in vivo studies. This is a result of the ability of 3D models to better mimic tumor heterogeneity, pH variation, nutrient and drug access, and cell-cell and cell-extracellular-matrix interactions [159,160].

To date, numerous 3D models of colorectal cancer have been developed with increased complexity, ranging from spheroids, composed of either one cell line or a co-culture, to the development of patient-derived organoids [161,162,163]. Spheroids are 3D cellular aggregates that can be produced using scaffold-free methods, such as the hanging drop method, cell culture on low-attachment plates, and forced-floating and agitation methods [161,162,163]. In addition, scaffold-based methods can be used, whereby cells are embedded in ECM constituents, such as collagen, hyaluronic acid, or mixtures such as Matrigel [161,162]. Recently, the need to obtain more complex and close-to-real 3D models led to the development of organoids. These are derived from pluripotent or multipotent stem cells that self-organize into 3D structures, which more closely mimic the anatomical and physiological features of an organ. One of their greatest advantages is the possibility of using patient-derived stem cells, leading to the development of personalized therapies [163,164].

**Figure 6:**
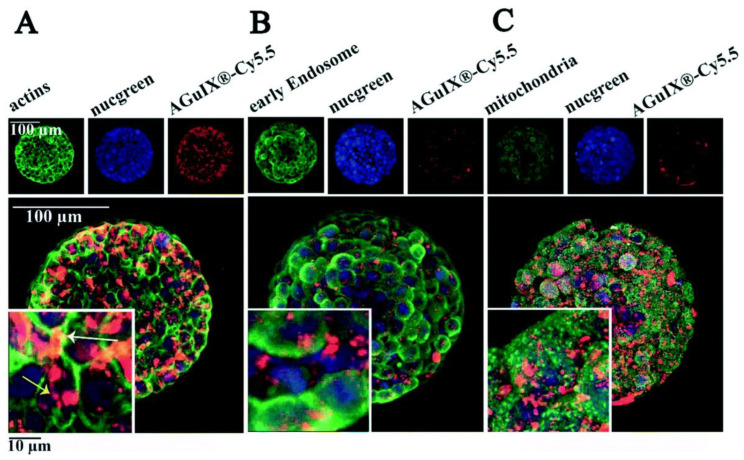
Penetration of polysiloxane and gadolinium chelate nanoparticles marked with fluorescent probe Cy5.5 (AGuIX -Cy5.5) in HCT-116 colorectal cancer spheroids and co-localization with different cellular structures: actin (**A**), early endosome (**B**), and mitochondria (**C**). Blue channel: cell nucleus; red channel: AGuIX -Cy5.5; green channel: phalloidin (**A**); EEA1 antibody-labeled early endosomes (**B**); AIF antibody-labeled mitochondria. Adapted with permission from [166]. Copyright 2022, Creative Commons.

Different studies have developed 3D models of colorectal cancer to better evaluate the toxicity and therapeutic potential of nanoparticles to be used as radiosensitizers. Hau et al. developed a combinatorial strategy based on spherical PEGylated gold nanoparticles (10 nm) and radiotherapy to enhance the treatment efficacy in colorectal cancer. Gold nanoparticles were chosen, as they can enhance the delivery of the radiation dose in the tumor area. Their toxicity was evaluated in both 3D ring models and 3D spheroids of colorectal cancer using the human colorectal cell line LOVO. Three-dimensional models were produced by incubating LOVO cells with Nanoshuttle, followed by aggregation by levitation using a magnet drive overnight in an ultralow-attachment 96-well plate. Nanoparticles were cytocompatible up to concentrations of 25 µg/mL, with 3D models being less susceptible to treatment than 2D models, demonstrating the importance of using the former to assess the toxicity of nanoparticles. Finally, a synergistic effect between gold nanoparticles and ionizing radiation (either kilo- or megavoltage) was observed in LOVO cells using the clonogenic assay [165]. A study aiming to combine small nanoparticles and radiotherapy was also carried out by Goodarzi et al. In this work, sub-5 nm nanoparticles composed of polysiloxane and gadolinium chelates were produced, and their penetration capacity was evaluated on multicellular spheroids of the human colorectal cancer cell line HCT-116. These nanoparticles are potent radiosensitizers and are currently in clinical trials in combination with radiotherapy for the management of different types of cancer. The authors developed a protocol for spheroid preparation using an agarose-based microsystem and performed a detailed study on the penetration, cellular internalization, and organelle distribution of the nanoparticles at different time points and concentrations (Figure 6). These data provide useful information to more effectively study the interaction and distribution of nanoparticles in a three-dimensional environment for future radiosensitization studies [166].

The efficacy of photothermal therapy has also been evaluated in different 3D colorectal cancer models. McCarthy et al. produced Hybrid Donor-Acceptor Polymer Particles (HDAPPs) composed of fluorescent and photothermal polymers and nanoparticles containing only the photothermal polymer (BSe). Nanoparticles (185 nm) were coated with hyaluronic acid (HA), and their interactions and photothermal ablation effects were evaluated in 3D organoids of colorectal cancer. Organoids were produced by mixing the mouse colorectal carcinoma cell line CT26 with a solution of extracellular matrix components (heprasil and collagen solution), followed by cross-linking through UV irradiation. The nanoparticles induced a time-dependent temperature rise after exposure to a continuous laser at 800 nm at 5 W, with BSe nanoparticles demonstrating the highest temperature rise (of more than 60 °C). A reduced penetration capacity of the HA-coated nanoparticles across the organoid was detected, with a higher accumulation in the organoid periphery, in comparison with their non-coated counterparts, in opposition to what occurred in 2D models. This could be related to greater interactions between the nanoparticles and the extracellular matrix. Nevertheless, HA-coated BSe presented the most effective photothermal ablation effect upon laser irradiation for 60 s, achieving almost the full eradication of the organoid. These results highlight the relevance of using 3D models to assess both the penetration and photothermal effects of nanoparticles, as 3D organoids required higher doses and longer incubation and irradiation times in comparison with 2D models [167].

Fiorica et al. developed graphene oxide-embedded nanogels composed of HA and polyaspartamide (300 nm) for the photothermal therapy of colorectal cancer. To improve the efficacy of the treatment, the chemotherapeutic drug irinotecan was incorporated into the nanosystem. Three-dimensional tumor models were obtained by seeding human dermal fibroblasts and HCT-116 cells in Matrigel. The nanosystem induced a time-dependent temperature increase upon irradiation with an 810 nm diode laser (3 × 10^−3^ W mm^−3^) for up to 150 s, demonstrating a ∆T of approximately 0.011 °C W^−1^ mg^−1^ and a mass extinction coefficient of 48.2 L g^−1^ cm^−1^. After the injection of the nanosystem into the 3D model and irradiation for 200 s, a necrotic spot was observed, which was enhanced in the presence of irinotecan, demonstrating the efficacy of the thermoablation treatment in combination with chemotherapy [146]. Roma-Rodrigues et al. also designed a strategy combining photothermal therapy and chemotherapy for colorectal cancer using spherical PEGylated gold nanoparticles (18 nm). The authors produced 3D spheroids of doxorubicin-sensitive and resistant colorectal cancer cell lines (HCT116 and HCT116-DoxR, respectively) by culturing the cells in super-low-attachment 96-well plates, followed by orbital shaking and incubation for 7 days. Tumor spheroids were then incubated with the nanoparticles and irradiated for 1 min with a diode-pumped solid-state laser (532 nm) coupled to a 1 mm diameter optical fiber at 3.78 W cm^−2^. A time-dependent loss of integrity of the tumor spheroid was observed for both 3D models. In addition, real-time data demonstrated that photothermal therapy improved the penetration and therapeutic efficacy of doxorubicin in both colorectal 3D models, demonstrating the relevance of combining localized photothermal therapy and chemotherapy to reduce chemotherapeutic drug doses and enhance the efficacy in cases of tumor drug resistance [168].

Overall, these studies demonstrate that there are large differences in the response to both radiotherapy and photothermal therapy between 2D and 3D models, highlighting the importance of using the latter to better assess treatment outcomes in vivo and in future clinical applications.

### 6.3. In Vivo Studies

In vivo studies of plasmonic gold nanoparticles (GNPs) of different sizes and shapes have already been performed for concomitant phototherapy and radiotherapy applied to colorectal cancer; however, combined photothermal and radiotherapy can also be chosen in future studies [110]. Most studies use nanoparticles with sizes that fall within 10–100 nm, as nanoparticles with a size below 10 nm are rapidly excreted by the kidneys. In comparison, those with a size above 100 nm are quickly captured by the immune system [169]. Different shapes have been used for in vivo studies, such as spheres [170], which present higher cellular uptake [110]; nanorods [171,172,173], which present high absorption coefficients [174]; and nanocages [6] or half-shells [130] for drug delivery.

Although there exist clinical trials that have investigated the applicability of GNP-based radiotherapy and phototherapy [98,175], current in vivo studies applied to colorectal cancer are limited to preclinical experiments [2]. Most preclinical models consist of xenograft models of human colorectal cancer cell lines injected subcutaneously into the flanks of mice [116,176]. In these models, the tumor is exposed, facilitating the penetrability and absorption of the radiation beam by nanoparticles distributed within the tumor [98]. For phototherapy, more clinically realistic models rely on tumors implanted in deeper organs such as the liver, requiring a catheter carrying a fiber-optic to irradiate the tumor [142,169]. Phototherapy studies make use of a NIR laser (808 or 810 nm [173]) to maximize penetration within the tumor, with irradiation times ranging from 2 to 10 min and laser power ranging from 0.5 to 12 W to achieve heating above 55 °C and induce thermal cytotoxicity [177], while radiotherapy studies make use of doses usually ranging between 4 and 30 Gy. The optimal laser exposure in phototherapy is a compromise between the photothermal conversion efficiency of the specific nanoparticle type [6], its temporal heating curve [122], and the integrity of the healthy tissue surrounding the tumor, as excessive laser exposure may cause undesired damage and apoptosis [121].

The efficacy of phototherapy and/or radiotherapy also depends on the proportion of nanoparticles accumulated within the tumor at a specific dose [121,178]. GNPs are deposited in the tumor upon systemic administration owing to the EPR given by fenestrations in immature blood vessels in the tumor [121]. However, the rate of deposition in the tumor is low [169], and GNPs accumulate in undesired organs of the reticuloendothelial system [121] regardless of their size, while small-sized GNPs accumulate in the rest of the tissues [177]. Several studies claim that the non-specific biodistribution of GNPs does not cause systemic toxicity during photothermal therapy [6,172,179] or radiotherapy [170,180] and that the renal clearance of GNPs is achieved 72 h after intravenous injection [181]; however, the toxicity of GNPs remains unclear and requires further studies [98,182]. Different strategies have been developed to maximize the aggregation of GNPs within the tumor and minimize potential systemic toxicity. These include GNP surface functionalization with hypoxia biomarkers such as CAIX [124], mesenchymal stem cells (MSC) [171], tiopronin [183], or antibodies such as the anti-death receptor-4 (DR4) monoclonal antibody [130] or the PD-ligand-1 antibody [6].

However, in radiotherapy, surface functionalization can have adverse effects due to the scavenging of low-energy electrons [101]. Other strategies involve the use of the pH-mediated aggregation of Au-RRVR complexes to increase tumoral aggregation [178]. The site-selective injection of theranostic GNPs is an invasive although effective alternative [169,176,183]. Theranostic nanoparticles enable both treatment and diagnosis by medical imaging [184] and are usually composed of a contrast material such as iron-platinum (FePt) [184] or gadolinium [169]. Photothermal therapy with GNPs is a promising treatment regimen for colorectal cancer. Many studies have demonstrated an evident recession of the tumoral volume [124,130,172,173,178,184,185]. Others have shown a significant increase in survival time [121,179] and even total survival [6,171], with minimal effects on surrounding healthy tissues. Similarly, GNP-based radiotherapy has shown promising results regarding tumor growth delay [180,183] and the survival rate, with up to 100% survival after 60 days [170]. The application of hyperthermia before radiotherapy produces an increase in perfusion that reduces hypoxia within the tumor, facilitating the radionecrosis of hypoxic-resistant tumoral cells [186]. Several clinical trials were conducted in the 1980s and 1990s to assess the viability of combined radio- and phototherapy, known as thermoradiotherapy [175]. However, the results were not promising due to the difficulty of achieving localized hyperthermic temperatures and the procedures’ invasiveness [186]. With the rise of nanotechnology, thermoradiotherapy studies have been reinitiated, although, to our knowledge, very few have yet been applied to colorectal cancer [186].

## 7. Conclusions and Future Perspectives

CRC is the third most prevalent cancer worldwide. Despite recent developments in the treatment of this pathology, which include a personalized approach using radio- and chemotherapies in conjunction with cutting-edge surgical techniques, it is critical to improve the effectiveness of these interventions and lessen their negative side effects on patients’ health. The use of NPs as radiosensitizers to enhance the responsiveness of cancerogenic tissues to radiotherapy has been researched throughout the past few years. The study of metallic, bimetallic, and metal-oxide-based NPs has drawn a lot of interest within this paradigm. In parallel, plasmonic-nanoparticle-mediated hyperthermia techniques, such as PTT, have been successfully carried out on animals with tiny tumors over the years, demonstrating its ability to selectively destroy malignant tissue. Considering the complexity of larger and more clinically significant tumors, PTT as a stand-alone treatment might not be viable in terms of complete tumor removal without reoccurrence. As a result, researchers have been motivated to investigate alternative applications of PTT in conjunction with other therapies to concomitantly benefit from its advantages. Enhancing the efficacy of chemotherapy, releasing anticancer medications, inducing an immune response to immunotherapy, and acting as radiosensitizers are some examples of how such NPs can be successfully applied to obtain a synergistic effect between different therapies.

Regarding CRC specifically, this review addressed studies performed from single cells to in vitro (2D and 3D cell cultures), in vivo, and clinical trials. Despite the promising results obtained for both radiotherapy and PTT alone, the combination of PTT with radiotherapy for the treatment of this specific cancer has not yet been reported and is therefore a new research path that should be explored in the future. The radiosensitization effect of hyperthermia needs to be explored and quantified as an enhanced equivalent radiation dose. Both the temperature and time interval between radiation therapy sessions need to be correlated with cell death outcomes, and their effects need to be understood.

## Figures and Tables

**Table 1:** Summary of highlighted preclinical studies evaluating photothermal therapy mediated by gold nanostructures in colorectal cancer.

Nanostructure	Preclinical Model	Application	PTT parameters	Ref.
PEGylated gold nanorods	CT26 tumor xenografts in vivo	PTT	Percutaneous irradiation with optical fiber; 808 nm; 3.5 W; 3 min	[121]
Immunotargeted gold-iron oxide NPs	SW1222 and HT29 tumor xenografts in vivo	PTT, MRI imaging	Laser; 808 nm; 5 W/cm^2^; 20 min	[122]
Gold-coated SPIONs functionalized with thiol derivatives	HT29 cells in vitro	PTT, MRI imaging	LED; 820 nm; 0.7 W/cm^2^; 2–8 min	[123]
Gold nanorods conjugated with carbonic anhydrase IX	HT29 cells in vitro and tumor xenografts in vivo	PTT	Laser; 760 nm; 12 W/cm^2^; 2–3 min	[124]
Silica-gold nanoshells	CT26 tumor xenografts in vivo	Fractionated PTT	Laser; 807 nm; 1.2 W/cm^2^, 5 min	[125]
